# Radiation-Induced Cardiovascular Disease: A Clinical Perspective

**DOI:** 10.3389/fcvm.2017.00066

**Published:** 2017-10-26

**Authors:** Syed Wamique Yusuf, Bhanu Prasad Venkatesulu, Lakshmi Shree Mahadevan, Sunil Krishnan

**Affiliations:** ^1^Department of Cardiology, University of Texas MD Anderson Cancer Center, Houston, TX, United States; ^2^Department of Experimental Radiation Oncology, University of Texas MD Anderson Cancer Center, Houston, TX, United States; ^3^Department of Radiation Oncology, Division of Radiation Oncology, University of Texas MD Anderson Cancer Center, Houston, TX, United States

**Keywords:** cardiovascular disease, cancer, radiation, ischemic heart disease, biomarkers

## Abstract

Cancer survival has improved dramatically, and this has led to the manifestation of late side effects of multimodality therapy. Radiation (RT) to the thoracic malignancies results in unintentional irradiation of the cardiac chambers. RT-induced microvascular ischemia leads to disruption of capillary endothelial framework, and injury to differentiated myocytes results in deposition of collagen and fibrosis. Coexistence of risk factors of metabolic syndrome and preexisting atherosclerosis in addition to RT exposure results in accelerated occurrence of major coronary events. Hence, it becomes pertinent to understand the underlying pathophysiology and clinical manifestations of RT-induced cardiovascular disease to devise optimal preventive and surveillance strategies.

## Introduction

**Figure 1:**
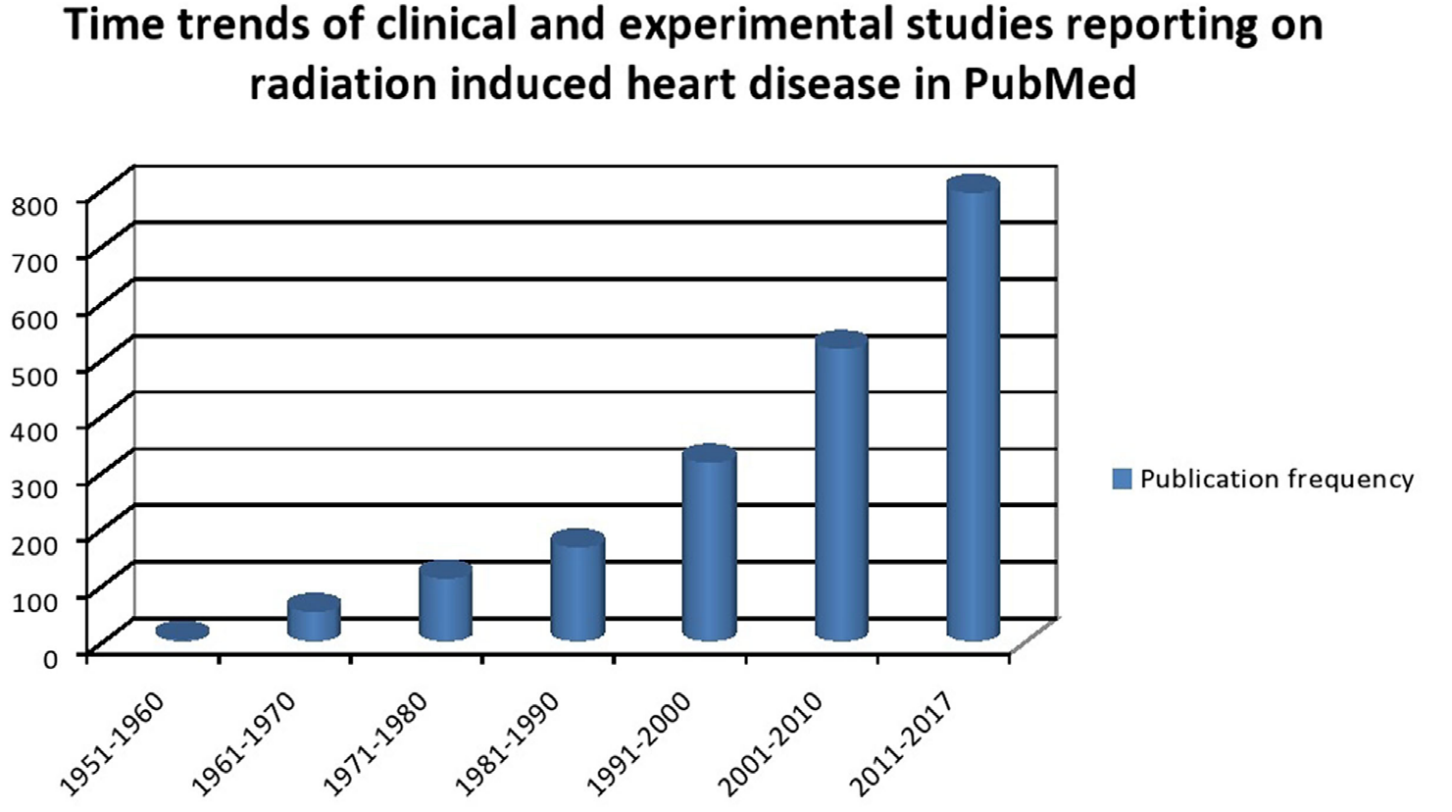
Time trends of clinical and experimental studies reporting on radiation-induced heart disease in PubMed.

Cardiovascular diseases are the number one cause of mortality worldwide with an estimated 17.7 million people dying of it. Cancer is the second most common cause of death worldwide in 2015 with an estimated 8.8 million cases (1). The heavy burden of CVS disease and cancer portends a scenario where the number of patients with coexisting cardiac disease and cancer is going to increase exponentially. Cancer management has undergone significant changes in the past century. Better understanding of tumor biology and advent of modern therapeutic arsenal has led to improved survival outcomes. As survival improves, there is superimposition of age-related chronic diseases with the chronic side effects of multimodality cancer therapy. It is estimated that over 50% of all patients diagnosed with cancer undergo RT either as a curative and/or supportive therapy (2, 3). Cancer survivors tend to have comorbidities such as diabetes, hypertension (HT), hyperlipidemia, chronic renal disease, and vascular diseases, which might be additive to RT in causing accelerated atherosclerosis or *vice versa* (4). Incidental RT dose to heart or vascular structure has been documented to produce long-term cardiovascular side effects. The risk of development of CV disease increases with increasing radiation (RT) dose to the heart and can cause structural and functional abnormalities of coronary vessels, valves, pericardium, and myocardium (5). Breast, lung, esophageal cancer, thymoma, and mediastinal lymphoma are the most commonly treated malignancies with RT that are in close proximity to heart and those that have high probability of being enclosed in the RT portal. Early-stage breast cancer and lymphoma have most of the best survival rates and hence have higher possibility of manifesting the late RT-induced cardiac side effects. Population-based cohort studies have shown that in long-term survivors of Hiroshima bombing, the incidence of vascular diseases such as coronary events and stroke are increased with linear increase in RT dose. In addition, chronic renal failure and liver cirrhosis are also increased, which may potentially influence the CVS outcomes (6). The literature on cardiac events in cancer patients are predominantly based on those treated between 1950 and 1990s and in an era of orthovoltage machines, primitive dosimetry techniques, extended RT fields, and high RT doses (7). This may not be truly reflective of the CVS risk current RT protocols imparts on cancer patients. Yet, even in the current era of advanced RT techniques such as image-guided radiotherapy, three dimensional dosimetry, and strict adherence of RT protocols dose effect association between RT and cardiac effect seems to exist (8). Analysis of time trends in clinical and experimental studies reporting RT-induced CV disease confirms the increasing interest in understanding the causes, effects, and prevalence of this phenomenon (Figure 1). As cancer survivorship and quality of life come into focus it is of paramount importance to understand the basic underlying mechanism of RT-induced cardiac dysfunction. In this review, we briefly outline the pathophysiology of RT-induced CV disease genesis and elaborate on clinical manifestations of CV disease in cancer patients who received RT, our current understanding of factors contributing to this, and propose strategies that can be undertaken to minimize this risk.

### Brief Overview of Pathophysiological Manifestations of RT-Induced Cardiovascular Injury

From an RT oncologist’s perspective, the heart is a serial organ as well as a parallel organ with the myocardium functioning as individual functional units whereas the vessels function as a single functional unit. Prominent pathophysiological changes noted in individual components of the cardiovascular system are briefly highlighted here.

Pericardial changes following RT are characterized by disruption of microvascular endothelial cells of the pericardium with repeated episodes of ischemia leading to fibrosis and formation of initial fibrinous exudates that are eventually replaced by fibroblasts and collagen (9). Endocardial changes following RT are most notable in the coronary vessels where ultrastructural changes in the capillary networks result in reduced capillary: myocyte ratio, damage to the epicardial vessels leading to upregulation of transforming growth factor-beta leading to a prothrombotic state, and activation of nuclear factor-kB leading to sustained inflammation (10–13). This may predispose to acceleration of atherosclerosis in view of increased recruitment of monocytes and macrophages to sites of active inflammation as well as vessel lumen occlusion secondary to prothrombotic milieu. The damage to the endothelium leads to migration of monocytes to the tunica intima and engulfment of lipoproteins with eventual formation of fatty streaks even in the absence of preexisting atherosclerosis (14). Risk factors such as hyperlipidemia appear to shorten the time to atherosclerosis development, as supplementing animals with fat diets increases the degree of atherosclerosis in rabbits undergoing RT, suggesting an additive effect of irradiation and other risk factors in producing RT-induced atherosclerosis (15). Japanese atomic bomb survivors had increased blood levels of pro-inflammatory cytokines like interleukin-6, C-reactive protein, and tumor necrosis factor-alpha suggesting an indirect association between chronic inflammation and RT-induced vascular damage (16). In experimental mice models with established atherosclerosis, RT tends to cause ultrastructural alterations in plaques leading to intraplaque hemorrhage, infiltration of macrophages leading to an unstable plaque that is vulnerable to thrombosis (17). The RT-induced inflammatory changes and the alterations in the endothelium act in cohesion to modulate the process of atherosclerosis in the coronary vessels. Hence, RT alone can initiate atherosclerosis and in addition can act to accelerate already established atherosclerosis. Myocardial changes following RT include myocardial fibrosis as a consequence of endothelial cell degeneration of myocardial capillaries. Direct myocardial injury is compounded by endothelial injury resulting in collagen deposition in the lumen of capillaries, stenosis of these vessels, and worsening myocardial blood supply creating a vicious cycle of reduced blood supply and continual fibrotic remodeling of the myocardium (18).

In addition to pericardial, myocardial, and endocardial injury, the cardiac valves and conduction system suffer from RT-induced injury as well. This may manifest as regurgitant valvular disease initially due to physical retraction of valves and stenotic disease later on due to fibrotic thickening, calcification, and valve retraction. Left-sided valves are found to be more frequently affected with one autopsy study showing diffuse valve fibrosis in 79% of mitral or aortic valves (19–21). RT-induced changes affect the vagus nerve/carotid sinus altering the baroreceptor reflex leading to elevated baseline heart rate (HR) and abnormal heart rate recovery (HRR) (22). Alternatively, a compensatory increase in the concentration of beta adrenergic receptors with increased stimulation of the sympathetic nerves resulting in autonomic dysfunction may occur following RT-induced myocardial injury (23). Diffuse fibrosis post-RT might lead to alteration of conduction pathways with associated fibrosis of sinoatrial node leading to rhythm changes and eventual complete heart block (CHB) (24).

#### Clinical Manifestations of RT-Related Heart Disease

Radiation can cause pericardial disease, ischemic heart disease (IHD), valvular disease, conduction system disease, autonomic changes, and cardiomyopathy (25, 26).

#### Pericardial Disease

Pericardial changes are the most frequent RT-induced CV disorder.

#### Acute Pericarditis

Acute pericarditis is a rare manifestation that may occur during or immediately after RT. Such is the rarity that even in high volume centers only eight RT-induced inflammatory pericarditis were reported over a period of 30 years (7). It presents with chest pain in the vicinity of RT therapy, in association with a rise in inflammatory markers such as neutrophil count and erythrocyte sedimentation rate. The electrocardiogram (ECG) may or may not show classic findings of pericarditis. Treatment is with non-steroidal anti-inflammatory drugs (NSAIDs) and colchicine. Steroid should only be used in resistant or cases unresponsive to NSAID, as its use is associated with high relapse of pericarditis (27). It is often benign and if it resolves with NSAIDs, RT need not be stopped. Patients who develop acute pericarditis should be followed up closely, since they have high risk of developing chronic pericarditis (28).

#### Delayed Pericarditis, with or without Tamponade

Chronic pericardial disease can develop months to years after completion of RT therapy and may present as large pericardial effusion. Accumulation of protein rich exudate in the pericardial sac may lead to pericardial effusion and the rapidity of accumulation may result in cardiac tamponade. Dyspnea, orthopnea, chest pain with clinical signs of distant heart sounds, hypotension, and distended jugular veins might serve as pointers for diagnosis. Echocardiography remains the gold standard for definitive diagnosis and to rule out a tamponade. Fukada et al. reported that mediastinal RT field width of >8 cm for esophageal irradiation was associated with increased incidence of pericardial effusion (29). Wei et al. in a series of inoperable carcinoma esophagus treated with chemo radiotherapy reported that the median time for onset of pericardial effusion was 5.3 months (range, 1.0–16.7 months). The dose volume parameter V30 (volume of pericardium receiving 30 Gy) more than 46% was associated with 73% pericardial effusion rate at 18 months post-therapy compared with 13% when V30 was less than 46% (*p* = 0.001) (30). RT-induced pericardial effusion should always be a diagnosis of exclusion with due emphasis on other causative factors. Most effusions are self-limiting, and cardiac tamponade requires emergency pericardiocentesis. Recurrent effusions might require a pericardiotomy.

#### Constrictive Pericarditis (CP)

Constrictive pericarditis is a long-term sequelae of any cardiac inflammatory pathology. Long-term survivors of pediatric Hodgkin’s lymphoma were found to have an incidence of 7% CP among 86 patients (10). Patients may present with intractable heart failure. Surgical removal of parietal pericardium is the treatment of choice for CP. Avgerinos et al. reported that over a 15-year period of 36 patients undergoing pericardiotomy for CP post-RT related were 8.3% (*n* = 3) (31). Bertog et al. reported of 163 patients who underwent pericardiectomy for CP and found that post-RT CP had the worst 7-year survival rate of 27% (95% CI 9–58) (32).

#### Coronary Artery Disease (CAD)

Researchers from Sweden examined the breast cancer and CAD registry and tried to correlate the RT portals and stenosis of coronary vessels. Left-sided breast cancer patients tended to have stenosis in the distal left anterior descending (LAD) artery and distal diagonal artery. In the analyses of women with breast cancer and RT hotspot areas [>15 mm volume outside the planning target volume (PTV) which receives dose larger than 100% of the specified PTV dose], the severity of coronary stenosis increased with the increase in the hotspot areas. Correa et al. assessed in 961 patients the pattern of CAD for early-stage breast cancer who received RT and subsequently underwent cardiac stress testing and/or catheterization for cardiovascular symptoms. At a median time of 12 years post-RT (range, 2–24 years), higher rate of stress test anomalies was found in the left (59%) versus right-side irradiated patients (8%) (*p* = 0.001). 70% of coronary changes were in the LAD artery (33). In a further analysis, the authors reported that mortality from any cardiac cause was 3.5% in left-sided vs. 2% in right-sided breast cancer patients. In the second decade post-RT, cumulative risk of cardiac deaths was 6.4% (95% CI, 3.5–11.5) for left-side vs. 3.6% (95% CI, 1.8–7.2) for right-sided patients. Chest pain, CAD and myocardial infarction were higher in left-sided radiated patients (*p* < 0.002). HT was associated with higher risk of CAD (34). Pooled analysis of dose-escalation studies in 127 patients in stage III non-small cell lung cancer showed that the incidence of 2- and 4-year rates of symptomatic cardiac events were 10 and 18%. The cohort who had CVS events had higher heart doses than patients without events (heart mean dose, 20 vs. 10 Gy; V5Gy, 56% vs. 34%; V30Gy, 29% vs. 12%, respectively). Baseline CAD was also higher in the cohort with major events (35 vs. 8%), which suggests that RT may contribute to acceleration of atherosclerosis (35).

Darby et al. reported from a population based surveillance study for major coronary events in 2,168 women post-RT and found that major coronary events had a linear dose response relationship with mean cardiac dose. Major coronary events in the first decade were 44% and 33% between 10 and 19 years and 23% greater than 20 years. Per gray increase in mean heart dose resulted in 7.4% (95% CI, 2.9–14.5; *p* < 0.001) linear increase in the incidence of major coronary events. Baseline existence of cardiac risk factors contributed to the magnified risk from irradiation (5). Left-sided irradiation resulted in higher coronary events compared with right-side irradiation. Women with preexisting IHD, diabetes, respiratory diseases, smokers, and circulatory diseases had higher risk of coronary events. Mean RT dose to the heart to ≥10 Gy resulted in 116% (95% CI, 59–195) increase in occurrence of major coronary events. The mean RT dose to heart was a better predictor of major coronary events compared with mean doses to LAD coronary artery (5). Early Breast Cancer Trialists’ Collaborative Group reported that the vascular mortality was significantly increased with RT (death rate ratio 1.30; SE 0.09) (36). Caution should be emphasized in interpretation of above mentioned data, since most of these patients had received RT with older modalities, with extended internal mammary fields, lack of standardized RT protocols and lack of advanced volume based dosimetry tools.

Clinical presentation for post-RT-induced CAD is variable and includes chest pain, dyspnea, heart failure, syncope, or even sudden death. Post-surgery somatic phantom pain, RT-induced skin fibrosis, post herpetic neuralgia, costochondritis, RT-induced fatigue, and reflux disease in post-RT esophagus patients are potential confounders that may mimic angina chest pain. Treatment principles of RT-induced CAD are similar to general population. However, if coronary artery bypass graft is required, bypass using internal mammary graft may not be possible in all cases, as this vessel may also be affected by RT (37). There is also a higher rate of restenosis in stented and bypassed vessels (38). Patients may have asymptomatic IHD, and this is frequently identified on stress testing (39).

### Valvular Heart Disease (VHD)

Valvular heart disease usually develops many years after completion of RT therapy. Patients irradiated >20 years before had increased incidence of aortic regurgitation (60 vs. 4%, *p* < 0.0001), tricuspid regurgitation (4 vs. 0%, *p* = 0.06), and aortic stenosis (16 vs. 0%, *p* = 0.0008) than patients within 10 years. Left ventricular fractional shortening and age- and gender-adjusted left ventricular mass was lower in irradiated patients (40). The risk of developing VHD in patient exposed to RT increases linearly with the RT dose. VHD rate correlated with the dose to the affected valve (*p* < 0.001) than to the prescribed mediastinal dose (*p* = 0.003) (41). Although majority of the patient have mild to moderate VHD, a close follow-up is essential as some patients may develop significant valvular disease, needing surgical or percutaneous intervention. Few reports have stated that the mean duration for an asymptomatic VHD to transform to symptomatic VHD is 5 years (20).

#### Cardiomyopathy and Congestive Heart Failure (CHF)

Exposure to high dose of RT leads to myocardial fibrosis. Diffuse myocardial fibrosis prevents the myocardium from functioning in unison and leads to systolic heart failure. The failing myocardium activates the renin–angiotensin–aldosterone mechanism and sympathetic overactivation resulting in ventricular remodeling which further exacerbates heart failure symptoms. The presentation may be similar to that of CP with effusion, but the symptoms are not resolved with fluid drainage or pericardial stripping (20). Left ventricular ejection fraction changes to the tune of 7–15% are found in patient’s treated with predominantly anterior weighted fields (42). Mulrooney et al. reported cardiac outcomes in a cohort of 14,358 5-year survivors as part of Childhood Cancer Survivor Study. They found that the children had a hazard ratio of 5.9 (95% CI 3.4–9.6; *p* < 0.001) for the incidence of CHF. RT exposure to heart of ≥15 Gy increased the hazard of CHF, MI, and VHD by two to six times in comparison with non-irradiated survivors. The risk of adverse cardiac outcomes persisted even up to 30 years of follow-up (43). Lind et al. reported that myocardial perfusion abnormalities were seen up to 6% in the LAD distribution compared with baseline. They found that percent irradiated left ventricle (*p* < 0.001), hormonal therapy (*p* = 0.005), and pre-RT hypercholesterolemia (*p* = 0.006) were factors associated with the perfusion defects (44). These subclinical perfusion defects may lead to microvascular ischemic changes leading areas of infarction leading eventually to fibrosis, and this is a slow process with latency and eventual cardiac function compromise.

#### Conduction System

In the acute phase, most patients have nonspecific ECG changes in relation to RT therapy. Gomez et al. reported that poor R wave progression and septal ST changes were the most common findings. RT-associated right bundle branch block is seen in patients who have undergone mediastinal irradiation due to close proximity of right bundle to the endocardium (45). Patients may rarely present with CHB years after completion of RT, and some of these patients may need a permanent pacemaker. Timeline for occurrence for CHB is variable ranging from 1–23 years post-RT (46).

#### Autonomic Dysfunction

Patients undergoing mediastinal RT are at risk of developing autonomic dysfunction. Groarke et al. tried to correlate the exercise capacity parameters in patients of Hodgkin’s lymphoma who received RT as part of treatment. Autonomic dysfunction parameters such as elevated resting HR (≥80 beats/min) and abnormal HRR at 1 min (≤12 beats/min if active cool-down or ≤18 beats/min if passive recovery) were elevated in irradiated patients. Incidence of autonomic dysfunction increases with RT dose and time as a factor. Elevated resting HR and abnormal HRR were associated with inferior exercise capacity. Abnormal HRR had a higher hazard of all-cause mortality (hazard ratio 4.60; 95% CI: 1.62–13.02) (22). Coexistence of DM may contribute to autonomic dysfunction, and angina chest pain changes may go unnoticed.

#### Pulmonary Veno-occlusive Disease

Pulmonary veno-occlusive disease is a rare accompaniment of post-RT and has been reported only in a few anecdotal case reports. Thrombus formation, fibrosis, hyperplastic lymphatics, haemosiderosis leads to alteration in the arterial and venular endothelium with arteriosclerosis and narrowing of vasculature may contribute to development of pulmonary HT (47).

#### Biomarkers of RT-Induced Cardiac Injury

Troponin T (TnT), troponin I (TnI), and brain natriuretic peptide (BNP) are potential biomarkers for cardiac injury. Gomez et al. did an exploratory study to assess TnI and BNP levels during RT for thoracic malignancies. The median BNP remained elevated for one patient post-RT (*p* = 0.042). BNP did not increase over time in the 18 patients who received only RT (45). Palumbo et al. tried to correlate BNP with cardiac dosimetry in left-sided breast cancer patients receiving adjuvant RT. BNP levels were elevated at (*p* < 0.001) 1 and 6 months after RT. Normalized BNP was significantly associated with V20, V25, V30, V45, mean heart dose, and maximum heart distance (*p* < 0.05). Four patients had coronary events and in the one patient with MI V20, V25, V30 and V45 were the highest, and BNP elevation was persistent from the 1–12 months of follow-up. BNP can serve as a surrogate marker for predicting post-RT cardiac events (48). TnT is an indicator of myocardial injury. Association of TnT levels with cardiac RT doses and echocardiography was assessed in a cohort of 58 breast cancer patients post-RT. TnT were elevated in 21% of patients during RT. Higher RT doses for the whole heart (*p* = 0.02), left ventricle (*p* = 0.03), volume of LAD artery receiving 15 Gy (*p* = 0.03), and 20 Gy (*p* = 0.03) were associated with elevation of TnT. Changes in the interventricular septum thickness and prolongation of the deceleration of ventricular contraction were also noted in irradiated patients (49). These studies only evaluated cardiac markers in the short term, whether it translates in predicting long-term outcomes remains to be elucidated.

## Surveillance Principles

**Figure 2:**
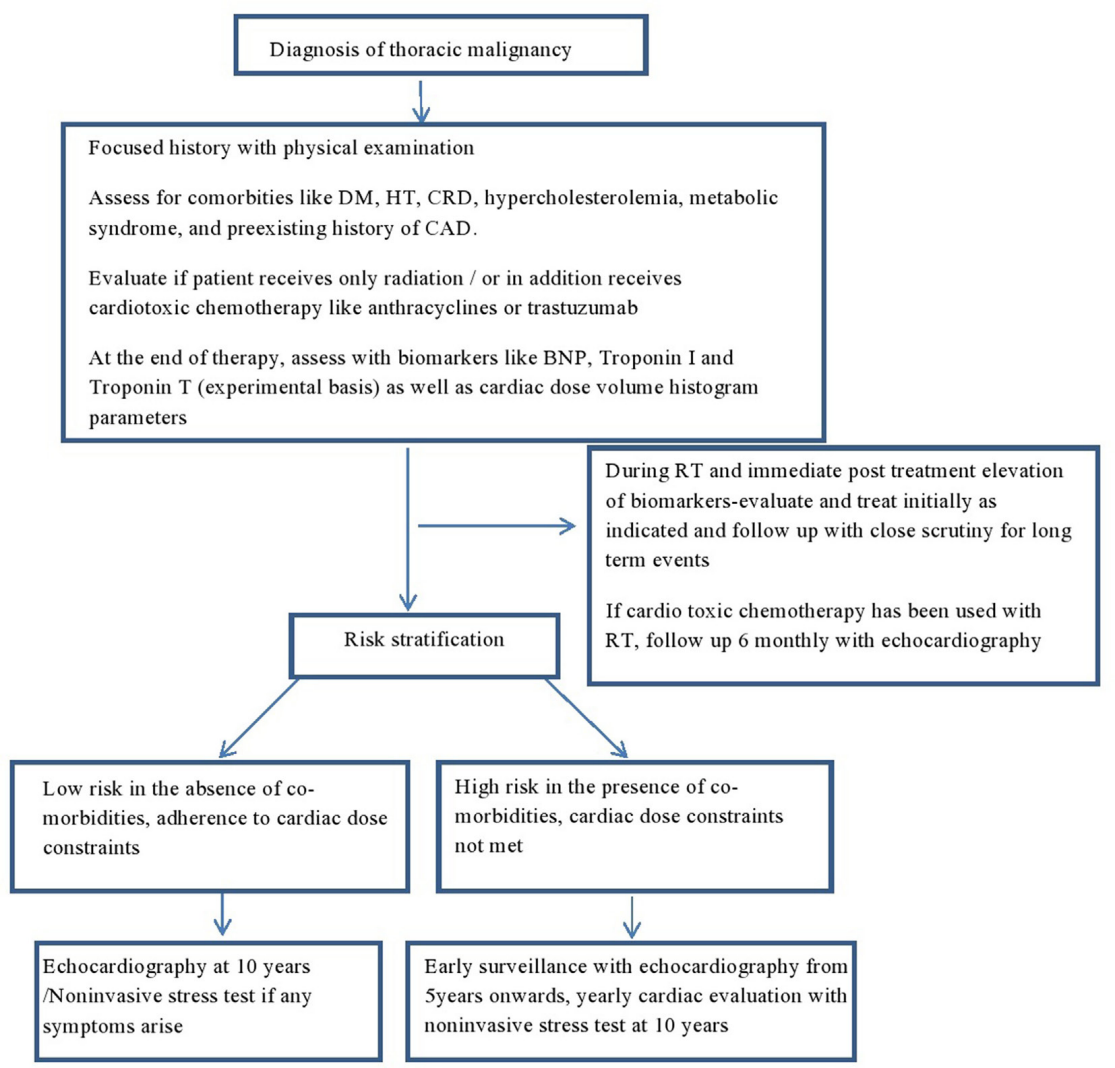
Proposed algorithm for follow-up in irradiated patients with thoracic malignancies. DM, diabetes mellitus; HT, hypertension; CRD, chronic renal disease; CAD, coronary artery disease; BNP, brain natriuretic peptide; TnI, troponin I; TnT, troponin T; RT, radiation.

Patients who are exposed to mediastinal RT or RT in the vicinity of the heart should be followed regularly and lifelong. A careful baseline history and physical examination is mandatory. Any patient with murmur or clinical risk factor should have a baseline echocardiogram. In addition, all patients should have a baseline lipid and thyroid function tests. There is no large scale data to support any specific pattern of clinical or imaging follow-up, but it reasonable to do a yearly clinical follow-up, and in asymptomatic patients a follow-up echocardiogram at 5 years in high risk patients and at 10 years in rest of the patients with a functional non-invasive stress test at 5–10 years in the high risk group. Another approach would be to obtain an echocardiogram at 5 and 10 years only if heart-related symptoms or murmur and consideration of stress test or CT scan of coronary artery after 10 years following mediastinal RT. At all follow-up, a careful history and physical examination is crucial. As some patients may develop asymptomatic pericardial effusion, the chest X-ray and CT scans that are frequently and routinely done in these patients for follow-up and staging should be carefully reviewed on each visit. A proposed algorithm for follow-up is illustrated in Figure 2.

## Conclusion

Cancer and cardiovascular disease are bound to present or develop as the life span of people keeps improving. Although most of the published studies on RT-associated cardiovascular events were from the older era of RT technology, some potential risk still persists as evidenced from the dose-escalation studies from lung cancer. Awareness of its underlying pathophysiology and the myriad of manifestations is required to detect and rehabilitate this cohort of patients. Risk prediction models and scrutiny of potential biomarkers may serve to predict the subset of patients who may develop cardiac events in the distant future. Heightened awareness of this complication following RT should result in adoption of careful surveillance programs during post-RT follow-up of patients. Increasingly, this will require close collaboration between RT oncologists, internists, radiologists, and cardiologists wherever possible. In an ideal program, a centralized registry of RT-induced CVS disease incidence and prevalence would be maintained at cancer centers in a manner similar to the tumor registry and would enlist active participation of oncologists, cardiologists, and radiologists.

## Author Contributions

Conception or design of the work, drafting the work, and final approval of the version to be published—SY, BV, LM, and SK. Agreement to be accountable to the accuracy or integrity of any part of the work are appropriately investigated and resolved—SY.

## Conflict of Interest Statement

The authors declare that the research was conducted in the absence of any commercial or financial relationships that could be construed as a potential conflict of interest.
